# Smallholder farmer resilience to extreme weather events in a global food value chain

**DOI:** 10.1007/s10584-023-03586-1

**Published:** 2023-10-30

**Authors:** William J. Thompson, Varun Varma, Jonas Joerin, Solhanlle Bonilla-Duarte, Daniel P. Bebber, Wilma Blaser-Hart, Birgit Kopainsky, Leonhard Späth, Bianca Curcio, Johan Six, Pius Krütli

**Affiliations:** 1 Sustainable Agroecosystems Group, Department of Environmental Systems Science, ETH Zurich, Switzerland; 2 Nature-based Solutions Initiative, Department of Biology, University of Oxford, Oxford, UK; 3 Department of Biosciences, University of Exeter, Exeter, UK; 4 Intelligent Data Ecosystems, Rothamsted Research, Harpenden, UK; 5 Future Resilient Systems, Singapore-ETH Centre, Singapore, Singapore; 6 Instituto Tecnológico de Santo Domingo (INTEC), Santo Domingo, República Dominicana; 7 School of Biological Sciences, University of Queensland, Brisbane, Australia; 8 System Dynamics Group, Department of Geography, University of Bergen, Bergen, Norway; 9 Transdisciplinarity Lab, Department of Environmental Systems Science, ETH Zurich, Switzerland

**Keywords:** Food system, Climate resilience, Smallholder, Trade, Extreme weather

## Abstract

Extreme weather events have severe impacts on food systems, especially for smallholders in global food value chains (GFVCs). There is an urgent need to understand (a) how climate shocks manifest in food systems, and (b) what strategies can enhance food system resilience. Integrating satellite, household and trade data, we investigate the cascading impacts after two consecutive hurricanes on smallholder banana farmers in Dominican Republic, and determinants of their recovery. We found that farmers experienced an ‘all-or-nothing’ pattern of damage, where 75% of flooded farmers lost > 90% of production. Recovery of regional production indicators took ca. 450 days. However, farm-level recovery times were highly variable, with both topographic and human capital factors determining recovery. Utilising this case study, we show that engaging in a GFVC impeded recovery via ‘double exposure’ of production loss and losing market access. Our results suggest that strategies to enhance resilience, with a particular focus on recovery, in GFVCs should promote trader loyalty, facilitate basin-scale collaboration and expand risk-targeted training.

**Supplementary Information:**

The online version contains supplementary material available at 10.1007/s10584-023-03586-1.

## Introduction

As the climate changes, smallholder farmers in tropical regions are increasingly vulnerable to extreme weather events, such as droughts, hurricanes and flooding (Harvey, et al. 2014; Dixon and Stringer 2015; Cottrell et al. 2019), which can cause loss of income, food insecurity and exacerbate environmental pressures (Morton 2007; Vogel et al. 2019; Parajuli et al. 2019). It is predicted that smallholders across multiple commodity value chains will face an increasing exposure to heatwaves, drought and heavy rains (Malek et al. 2022). Climate-driven impacts on smallholder farmers are often transmitted to the food systems they are part of, causing, for example, food price volatility and reducing food availability (Beer 2018; Holden and Shiferaw 2004; Venkat et al. 2022). Furthermore, this transmission of impacts can cross national boundaries and lead to cross-border climate vulnerabilities (Ercin et al. 2021). Hence, understanding how extreme weather events impact smallholder farmers and what determines their recovery is crucial towards building more resilient food systems (Fanzo et al. 2018).

Increasingly, many smallholder farmers are embedded in global food value chains (GFVCs), producing crops for export, including fresh fruit and vegetables (e.g. banana and beans), tropical commodities (e.g. cocoa and palm oil) and cereals (e.g. rice and maize) (Swinnen 2007). GFVCs are international networks of actors that interact at various stages (production, processing, distribution, retailing and consumption) of the food system (Ericksen 2008). However, there are several gaps remaining in our understanding of how farmers engaged in GFVCs are affected by climate shocks, and how the impacts are influenced by their participation in a GFVC (Donatti et al. 2018). Farmers participating in GFVCs may enhance their resilience with easier access to insurance (Isakson 2015), cooperative formation and price premiums (Sellare et al. 2020), but conversely, could lose from price exposure (Kaplinsky 2004) and crop quality pressures (Handschuch et al. 2013). Participation in GFVCs has also been suggested to create a double exposure to both climate and global market shocks (O’Brien and Leichenko 2000; Laube et al. 2012; Castellanos et al. 2013).

**Fig. 1:**
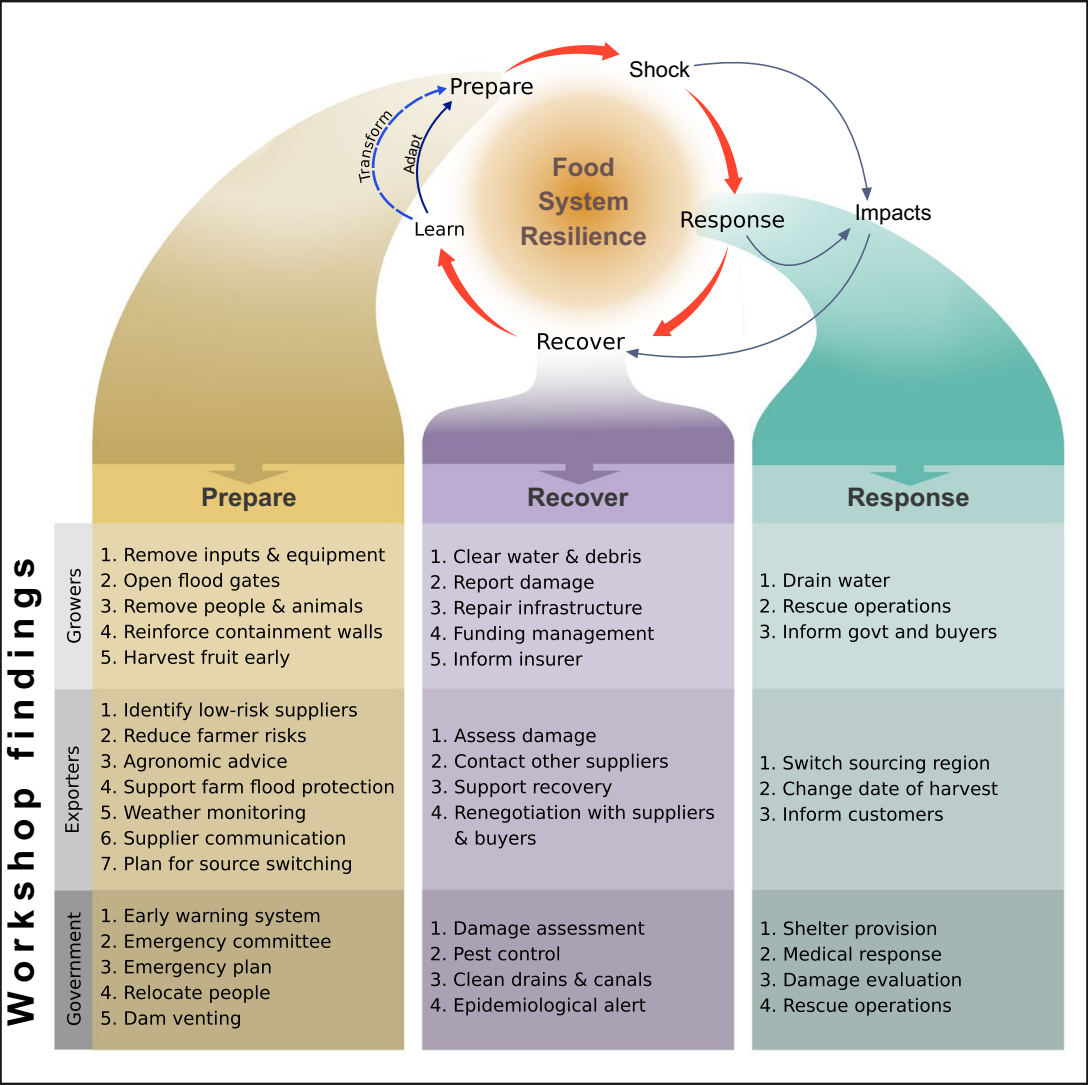
Banana GFVC actor activities to enhance resilience to hurricane shocks in the context of a food system climate resilience framework (developed from Tendall et al., 2015 and Thompson et al., 2022). The climate resilience framework consists of four strategic stages utilised by actors: (i) preparing for or (ii) responding to a shock, (iii) recovering from that shock and then (iv) learning to improve future outcomes, either by incrementally adapting or by fundamentally transforming the system. These results are from interviews and participatory activities in a workshop regarding extreme weather events held with banana GFVC actors

As awareness of the future risks that extreme weather events pose to smallholder farmers and our food system has grown, ‘climate resilience’ has emerged as a theoretical, governance and management approach to understand and reduce the impact of such shocks (Dixon and Stringer 2015; Tendall et al. 2015). In general, resilience is the ability of a system to maintain function, recover and, if possible, improve in the face of a shock (Holling 1973; Folke 2006) (Fig. 1). Here, as a result of our transdisciplinary stakeholder-led research process, we focused on recovery, the process of restoring livelihood systems to a normal or new functional state post-shock (UNISDR. Build Back Better. 2017). Recovery has been highlighted as critical in response to extreme weather events (Cottrell et al. 2019; Campbell and Beckford 2009) because faster recovery reduces the overall impact of shocks by restoring income generating assets and, thus, catalysing replenishment of non-productive assets (Carter et al. 2007). There has been limited research into the determinants of recovery for smallholder farmers from extreme weather events, though farm diversity and landscape topography have been suggested as potential influential factors (Alhassan 2020; Rosset et al. 2011a, 2011b; Philpott et al. 2008). Relatedly, in the wake of the COVID-19 pandemic, there has been increased research interest in the topic of resilience and recovery, including studies relating to food systems (Barman et al. 2021). Understanding the determinants of recovery in smallholder agricultural settings is critical to designing appropriate climate resilience enhancing strategies.

Strategies to enhance the resilience of smallholder-led GFVCs have been proposed and implemented at a range of scales by different food system actors, including farmers, cooperatives, non-governmental organisations, traders, retailers and governments (Ghadge et al. 2020). These actors use diverse governance mechanisms including national regulations, sourcing policies and certifications, as well as direct investments to implement their strategies (Prasad and Sud 2019; Garrett et al. 2021; Cohn et al. 2017; Stoll et al. 2021). These strategies include features, such as early warning systems, farm management training, premium payments, financial instruments for risk sharing and preparedness exercises (Boyd and Cornforth 2013; Deans et al. 2018; Kos and Kloppenburg 2019; Tadesse et al. 2015). Additionally, digital strategies that increase smallholder inclusion in GFVC decision-making have been posited to enhance their resilience (Quayson et al. 2020). Recent research has investigated the effectiveness of these GFVC strategies to enhance the climate resilience of smallholders, with mixed evidence citing strong influence on the adoption of agricultural technologies but weaker influence over key diversification strategies (Thompson et al. 2022). Beyond this, trade-offs with other societal goals, such as competition for land, and equity implications, must be considered when designing such strategies (Smith et al. 2020; Williams et al. 2020). A key research gap that remains is understanding how existing strategies perform in the face of real-world shocks, such as extreme weather events, and how the outcomes of such shocks can inform the design of future strategies.

In this study, we ask (i) how do extreme weather events impact food systems? (ii) How do the actors in GFVCs respond to extreme weather events? (iii) What determines the recovery of smallholder farmers embedded in GFVCs? We used a transdisciplinary research approach that provides the following contributions to the literature on food system climate resilience: (i) a novel integration of satellite and household data to study the dynamics of shocks to food systems, (ii) a new systemic perspective on the cascading effects of food system shocks and actor responses, (iii) new evidence on the consequences of smallholder farmers participating global food GFVCs, and (iv) identification of potential approaches to enhance the resilience of smallholder GFVCs. This approach of integrating household, remote sensing and trade data on the dynamics of shock responses has the potential to provide valuable insights when applied to other geographical areas and food systems.

## Methods

To address our research questions, we take the case of the GFVC connecting Dominican Republic (DR) banana production to the UK consumer market. This case is globally relevant as the banana GFVC typifies the challenges of smallholder GFVCs (Castillo et al. 2000; Riisgaard and Hammer 2011; Vagneron and Roquigny 2011a; Varma and Bebber 2019; Bebber 2019), specifically because the DR has a high dependence on agricultural exports, significant smallholder production (Vagneron and Roquigny 2011a), severe climate change exposure (Eckstein et al. 2020), and the GFVC is coordinated by powerful trade and retail organisations from the Global North.

This polarisation of power in food systems leads to the frequent inequitable framing of sustainability issues (Nelson and Tallontire 2014). Therefore, to integrate the stakeholders of the DR-UK banana GFVC into our research process, we adopted an overall transdisciplinary research methodology. Transdisciplinary research involves, inter alia, the co-defining of problems and co-generation of knowledge and solutions between scientists and stakeholders (Pohl et al. 2017; Lang et al. 2012; Pohl and Hadorn 2007). Throughout the research process, the research team critically reflected on the implementation of each activity, including how power dynamics between GFVC actors could influence participation in these activities. This included reflecting on how the position of the researchers in this system could be used to balance the range of stakeholder voices throughout the process (Chevalier and Buckles 2019).

We present four stages here: (i) co-defining climate risks in the DR-UK banana GFVC through semi-structured interviews and focus groups, (ii) hurricane shock and response characterisation for this GFVC through a stakeholder workshop, (iii) farm-scale resilience assessment through a survey of smallholder banana farmers and trade data analysis, (iv) national-scale recovery assessment through remote sensing of regional flood damage. We summarise the methodology interlinkages in Suppl. Mat. 1.

### Dominican Republic-UK banana value chain

#### DR banana production

**Fig. 2:**
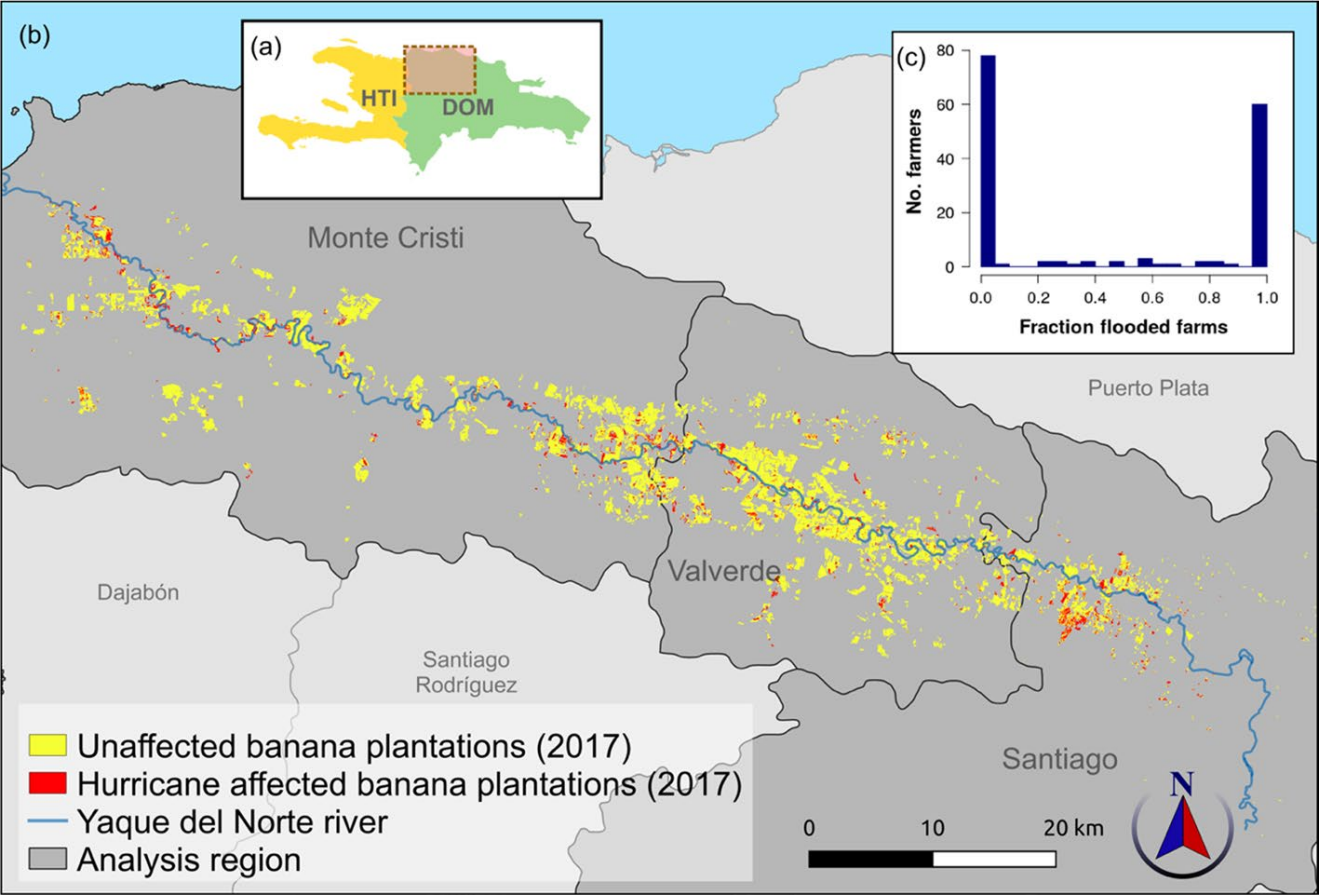
(**a**) Study area located in the northwest of the Dominican Republic identified by orange box. (**b**) Map identifying locations in the study region where banana farms in 2017 were affected by hurricanes Maria and Irma (September 2017). On overlaying the hurricane damage and banana farms maps for 2017, we estimate 2447 ha of farms were likely to have experienced damage. This accounts for 11.4% of area under cultivation before the hurricanes struck the Dominican Republic. (**c**) Histogram showing the number of farmers in our household survey experiencing different magnitudes of flooding, as represented by the fraction of their total farm area that was flooded. This demonstrates the ‘all-or-nothing’ nature of this shock

Bananas (*Musa acuminata Colla*) are a critical crop in the global food system, being in the top ten crops, in terms of cultivated area, production quantity and calories provided, according to FAO data. The largest exporting region is Latin America, which accounts for 80% of exports globally. The DR is the Caribbean’s largest banana exporter (FAOSTAT. Available at 2021) and the world’s largest organic banana exporter (Willer and Lernoud 2019). In addition, the DR faces several severe climate threats, including reoccurring droughts and tropical cyclones that cause significant damage through heavy rainfall, flooding and strong winds, as well as sea surges (IISD 2013). Export banana production in the DR is concentrated in the regions of Valverde and Monte Cristi (Fig. 2(a)). These provinces are dominated by the drainage basin of the Yaque del Norte River. The drainage basin has suffered from severe deforestation over the past decades (Sambrook et al. 1999). This has affected the regional hydrological regime, further exacerbating the scale of floods at times of heavy rain and reducing water availability during times of drought (Brandimarte et al. 2009).

#### DR-UK banana GFVC

Globally, banana production for export is carried out by a mixture of large plantations and smallholders. This varies by country, with the DR and Ecuador having a high proportion of smallholders. There are approximately 2000 banana farms in the DR, of which the majority (80%) are small scale (< 3 ha) and medium scale (3–10 ha) and a minority (16%) of the producers are women (EEAS, 2018). More than 300,000 people benefit directly or indirectly from the banana industry in the DR. The UK currently purchases around 60% of the DR banana production which accounts for around 17% of UK imports (Fair 2015). The smallholder farmers and workers that operate these farms are particularly vulnerable to climate shocks because they have a heavy reliance on natural resources and low adaptive capacity, due to low incomes, unstable commodity prices and wider poverty drivers (Rhiney 2015; Williams et al. 2018). Farmers’ vulnerabilities are also influenced by gendered-power relations within households, communities and the value chain (Gonda 2019).

#### Hurricanes Irma and Maria

On the 8th September 2017, Hurricane Irma, a category 5 hurricane, passed the northwest coast of the DR at a distance of 96 km. Wind speeds of up to 286 km h^−1^ and heavy rain caused severe damage to farms. The heavy rain led to severe flooding in the Yaque del Norte drainage basin. Just 7 days later, a second hurricane, Maria, also grazed the North West coast of the DR as a category 3 hurricane, bringing further flooding (Blake 2018).

### Co-defining climate challenges in the banana GFVC

In the first stage of our research, we collaboratively defined the specific challenges stakeholders in the banana GFVC face when extreme weather events strike. This was done to allow these stakeholders to guide the key focus of the study, thereby making it more relevant to their needs and avoiding preconceived conceptions of how a resilience approach could benefit them. To do this, we convened a platform of stakeholders from the DR-UK banana GFVC around the axis of smallholder producers in cooperatives in the DR supplying a major UK retailer, incorporating all the intermediaries and auxiliary actors in this GFVC (Suppl. Mat. 2).

To identify the full contingent of stakeholders for the study and climate-related challenges in the GFVC, we organised a series of semi-structured interviews with key actors from the DR-UK banana GFVC, including retailers (*n* = 1), importers (*n* = 2), exporters (*n* = 2) and banana farmer organisations (*n* = 4). Following this, we also arranged a series of focus groups with smallholder banana farmers in the DR (5 groups of between 5 and 8 farmers) to understand how climate shocks affect their livelihoods, and to decide which aspects would become the focus of the study. Through this process with the stakeholders, hurricane-induced flooding was identified as a major threat to the banana GFVC to investigate.

### Hurricane shock characterisation for the banana GFVC

In February 2019, we held a workshop with the identified GFVC stakeholders in Mao, Valverde, DR. The workshop had two key aims: (i) characterise the systemic structure of the banana GFVC to understand how shocks are transmitted and feedback within the system and (ii) characterise the actions that actors take to enhance their resilience to such shocks. To jointly characterise the systemic structure of the banana GFVC, we conducted a group system dynamic model building exercise, with each of the actors identifying key variables in their systems and connecting the variables that influence each other (Luna-Reyes et al. 2006). To understand the options available to different actors to enhance their resilience to flood shocks, we introduced the resilience framework that we developed based on Tendall et al. (2015) (Fig. 1). Using participatory research methods, including through focus groups, GFVC actors described what options are available to them in terms of preparation, response and recovery to a flood shock. A thematic analysis of these responses was performed, whereby stakeholder responses were coded and allocated across a matrix generated from our resilience theoretical framework (Williams et al. 2020). During the workshop process, stakeholders identified the recovery process after such extreme weather shocks had occurred as a key area to focus the study on.

### Resilience assessment

#### Questionnaire

Following the workshop, to investigate hurricane impacts and farmer resilience, including recovery, we conducted a survey of smallholder banana farmers within the DR-UK banana GFVC, in February and March 2019. The survey used a resilience assessment questionnaire based on the workshop outcomes and refined in focus groups with banana farmers, cooperatives and researchers. The digitised resilience assessment questionnaire contained six sections including household, agronomy, marketing, preparation, impacts, response and recovery. The English version of the questionnaire is included in Suppl. Mat. 15. The survey data was supplemented with GFVC data on prices and volumes from the UK wholesale market (Suppl. Mat. 3). Semi-structured follow up interviews with farmers were conducted in January 2020 to validate the results of the analysis (*n* = 12).

#### Sampling

For the household survey, in the Yaque del Norte drainage basin, we sampled farmers from four farmer cooperatives that supply two major exporters in the region, making up 28% of DR banana exports (O’Brien and Leichenko 2000). To understand the impact of the shock, we preferentially sampled farmers with farms in proximity to rivers (Suppl. Mat. 4). The sample consisted of 158 smallholder banana farmers engaged in the international export GFVC (Suppl. Mat. 5). Eleven percent of farmers were female with the mean age being 50 (standard deviation = 13).

#### Resilience assessment data analysis

To explain what influences the resilience, including recovery, of smallholder farmers after a hurricane shock has occurred, we analysed the data collected in the household survey using descriptive statistics, exploratory factor analysis and multiple linear regression.

To identify the factors that influence smallholder farmer recovery rates, we used exploratory factor analysis. We identified potential explanatory variables linked to the recovery process from extreme weather events, a priori, from the existing literature on smallholder resilience, focus groups with farmers and the workshop held with banana GFVC stakeholders (Suppl. Mat. 6). Using these variables, we conducted an exploratory factor analysis, following Field (Field 2013). First, to test issues with multiple collinearities in the explanatory variables, we conducted a correlation analysis using Pearson’s correlation coefficient on the 80 flooded households. Variables related to recovery time were selected based on significant correlation coefficients. Bartlett’s test of sphericity was conducted to determine whether factor analysis was suitable with regard to the relatedness of the variables (see in Field (2013)). The Kaiser-Meyer Olkin approach was used to determine sampling adequacy, based on common variance. Variables not meeting this criterion were discarded. Following this, an initial un-rotated principal component analysis (PCA) was conducted with these variables. The Kaiser criterion (> 1) was used to determine the number of factors to extract based on the eigenvalues of the unrotated components (Kaiser 1960). These components were thus extracted, and their axis rotated using VARIMAX technique. We then used multiple linear regression to assess the influence of these factors on recovery time. The analysis was carried out using the *psych* package with R statistical software (Revelle 2017).

For the exploratory factor analysis, the correlation analysis revealed nine potential explanatory variables significantly correlated with recovery time (including subcomponents replanting time and delay time) from the 23 tested (Suppl. Mat. 7). The initial PCA generated four components with eigenvalues greater than 1. Therefore, four components, explaining 79% of the variation, were extracted and rotated (Suppl. Mat. 8).

These four components were explored as drivers of recovery time with multiple linear regression using the factor scores of the four derived components. The following model was therefore formulated using the PCA components:

$$Recoverytime={\beta }_{0}+{\beta }_{1}Scaleofdamage+{\beta }_{2}Farm\wedge livelihooddiversity+{\beta }_{3}Floodtraining+{\beta }_{4}Drainage+\varepsilon$$

### Remote sensing for national-scale recovery assessment

#### Mapping banana production area and impact of hurricanes

Remote sensing analysis focussed on the three provinces of Monte Cristi, Valverde and Santiago. We mapped pre- and post-hurricane banana plantation area in the three provinces at a 10 m × 10 m resolution using a fusion of Synthetic Aperture Radar (SAR; Sentinel-1), multi-spectral (Sentinel-2) and terrain data (Shuttle Radar Telemetry Mission; SRTM). A random forest classifier for 2019 (post-hurricane) banana production area (accuracy of 99.8%) was trained using 100 ground truth plantation polygons for the same time period, and the classifier was projected to satellite imagery for the period of 2017 to map pre-hurricane production area. To map hurricane damage to plantations, SAR data from Sentinel-1 from up to 12 months before and 3 months after the hurricanes were used. Three types of damage were mapped, i.e. (1) open flooding—represented by a large decline in backscatter in the Sentinel-1 imagery immediately after the hurricanes; (2) a 100-m buffer around pixels identified as open flooding (to account for inundation that is obscured by standing vegetation); and (3) a legacy effect—identified as a negative deviation in pixel backscatter values after the hurricanes relative to its average value for 12 months prior to the hurricanes. A spatial union of these three components represented pixels that had experienced hurricane damage. A brief summary of these methods is provided in Suppl. Mat. 9, and are available in detail from Varma et al. 2020.

#### Quantifying recovery of banana plantations

Pixels which were classified as banana plantations in both 2017 (pre-hurricanes) and 2019 (post-hurricanes) were subsetted to estimate time to recovery. The pixels were categorised as having been either affected or unaffected by the hurricanes. We estimated time to recovery using two metrics. For a more lenient metric, a set of 6500 random sampling points were generated within each group, such that minimum spacing between points was 50 m. Sentinel-1 VV polarisation backscatter values, averaged within a 50 m × 50 m window, were extracted at each sampling point from every Sentinel-1 image available from March 2017 to April 2018 (68 images). The spatial averaging in a 50 m window was conducted to eliminate speckling artefacts that SAR data suffers from when working at fine spatial resolutions. Separately for the flooded and non-flooded pixels, we calculated the first quartile (Q1), median (Q2) and third quartile (Q3) of the VV backscatter values across the study region for every date that Sentinel-1 data were available for. These data (i.e. Q1, Q2 and Q3) were visualised as a function of date of image capture to illustrate the deviation in backscatter values in flooded pixels after the hurricane events relative to non-flooded pixels.

The second, more stringent approach, calculated a Productive Farm Index (PFI) as a surrogate for the proportion of plantation area that was in a productive state. The rationale for this analysis is that for non-flooded pixels, by definition, 75% of pixel values should be greater than or equal to Q1 of all non-flooded pixel values. Prior to the hurricanes, 75% of subsequently flooded pixels should also show values greater than or equal to Q1 of non-flooded pixels (i.e. Q1 of flooded pixels ≈ Q1 of non-flooded pixels). A loss of structural complexity following the hurricanes (through direct hurricane damage or clearing of affected plants after the hurricanes) leads to lower backscatter values in flooded pixels, which in turn results in less than 75% of pixels with values greater than or equal to Q1 of non-flooded pixels. The deviation in the fraction of flooded pixels which meet this criterion can be used as a proxy for the fraction of affected pixels that are not in a ‘productive state’. As post-hurricane recovery of production area progresses, and more affected area returns to a productive state, the fraction of flooded pixels with values greater than or equal to Q1 of non-flooded pixels will increase. This will continue until, once again, Q1 of flooded pixels ≈ Q1 of non-flooded pixels, and recovery is said to be completed. The PFI was obtained by first subsetting the sampled flooded pixels, such that only pixels whose VV backscatter values were greater than or equal to Q1 of non-flooded pixels for a minimum of eight out of the 15 image dates prior to the hurricane events were retained. This subsetting step minimised large fluctuations in backscatter values between consecutive images from having a disproportionate influence on the analysis, and is primarily observed in pixels at plantation edges. In total, 3365 flooded pixels were retained for this analysis. Then, for each date, the proportion of flooded pixels with a backscatter value greater than or equal to Q1 of non-flooded pixels for the corresponding date was calculated. Recovery time was calculated from the onset of the first hurricane event till the date when at least 75% of flooded pixels first showed a backscatter value greater than or equal to Q1 of non-flooded pixels (Suppl. Mat. 10).

## Results

### GFVC-actors use diverse strategies to reduce hurricane impacts

From analysis of our workshops and interviews, 18 months after the flood events, we found that DR-UK Banana GFVC stakeholders, including smallholder farmers, importers, exporters and the DR government, take a variety of actions in preparation for, response to and recovery from the hurricane-induced flooding (Fig. 1). In terms of preparation, smallholder banana farmers reported having a limited range of actions available to reduce the direct impact of flooding on their farms. For example, actions such as reinforcement of containing walls, to prevent inundation, were perceived to be of low efficacy. Importers and exporters took less direct action related to their activities and performed more coordinating actions leading up to the hurricanes. This included avoiding purchasing from high-risk farmers, but in contrast, also working with farmers to reduce flood risk by supporting the establishment of buffer zones near water sources. The Ministries of the Environment and Natural Resources reported taking two key actions in preparation for a hurricane in relation to the banana GFVC: preparation of a disaster response plan, and consequently, damage limitation activities involving relocating people from vulnerable areas and dam venting.

Responses following the start of flooding in September 2017 were enacted by these GFVC actors at multiple scales, including farm, watershed, nationally and internationally. Farmers reported taking key damage limitation actions, such as rescue operations for people and livestock, as well as communicating loss of production to buyers. Importers and exporters took two key types of action ‘switching sourcing location’ and ‘communication’ to inform buyers. This resulted in the retailer in this value chain reporting no consequences to their banana availability as a consequence of this shock. Government responses focussed on saving lives through rescue operations and provision of shelters.

Analysis of household survey data revealed that the adoption of resilience-enhancing strategies was relatively uniform across farmers that directly experienced flooding in 2017 versus those that did not (Suppl. Mat. 11). These included crop diversification (mean number of crops farmed = 2), intercropping (40% practising), income diversification (41%), training in flood damage prevention (56%) and insurance (23%). Insurance was the only strategy for which there was a significant difference between farmers that were flooded in 2017 and non-flooded farmers, with 36% flooded farmers adopting versus 9% non-flooded (chi-square 21.217, *p* < 0.01). However, insurance adoption of 36% amongst flooded farmers is still relatively low, with farmers citing cost and trust in the scheme as the main concerns. This compares, for example, with 63% of export banana farmers adopting weather insurance in the Windward Islands (Carballo Reis 2013).

### ‘All-or-nothing’ damage makes recovery key to farmer resilience

Analyses of Synthetic Aperture Radar (SAR) imagery revealed that 2447 ha or 11.4% of banana production area in the three regions were affected by hurricane related damage, and largely concentrated around the Yaque del Norte river (Fig. 2(b)). This estimate includes damage caused by open-water flooding in the immediate aftermath of the hurricanes, as well as more protracted storm damage over a period of 3 months since the hurricanes. In our survey of 158 banana farmers, 80 (51%) reported being directly impacted by hurricane-induced flooding. Of these flooded farmers, 75% (60 out of 80 farmers) reported 90% of their production area flooded (Fig. 2(c)). This suggests an ‘all-or-nothing’ nature of storm damage, i.e. when farms are affected, damage is complete and catastrophic. No farm-scale strategies were reported that were able to prevent this damage.

**Fig. 3:**
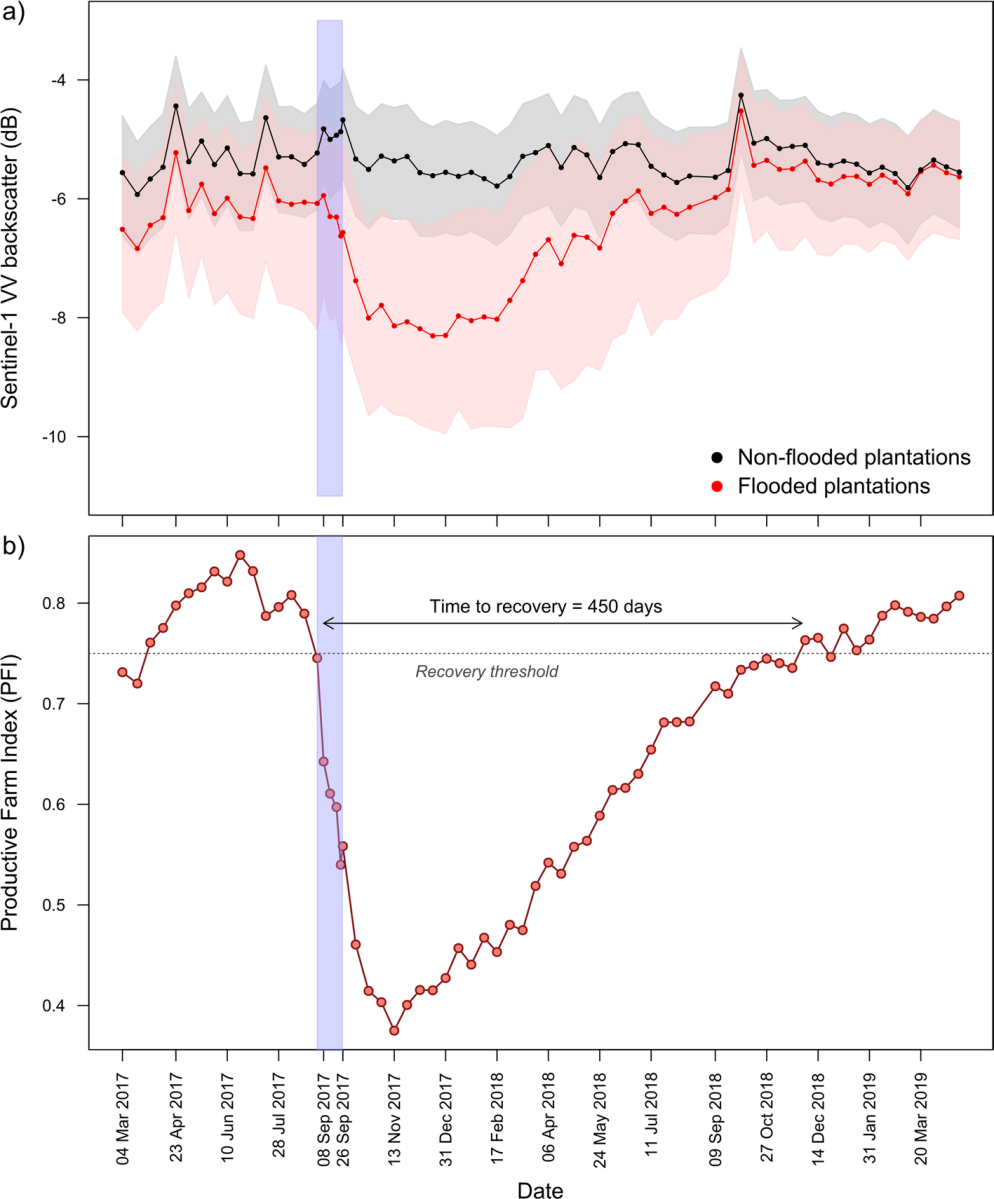
Impact and recovery assessment of banana production systems in the Dominican Republic using remote sensing. (**a**) A timeline of banana plantation canopy structure as indexed by the Sentinel-1 VV polarisation band. The solid lines represent median VV backscatter values for flooded and non-flooded banana plantation pixels sampled in the study area. Shaded areas around the solid lines represent the bounds of the 1st and 3rd quartiles. The blue shaded area indicates the date range when hurricanes Irma and Maria struck the Dominican Republic. (**b**) A timeline of the Productive Farm Index (PFI) for banana plantations from March 2017 to April 2019 in the study region. The blue shaded area indicates the date range when hurricanes Irma and Maria struck the Dominican Republic. The PFI represents the fraction of sampled banana pixels that were affected by hurricane damage with a Sentinel-1 VV polarisation backscatter value greater than, or equal to the 1st quartile of backscatter values from unaffected banana plantation pixels. Recovery from the hurricanes is assumed to be completed when the PFI value is 0.75 or above. This method estimates production in hurricane affected pixels reached pre-hurricane capacity approximately 450 days after the hurricane events

The flood waters (Photographs in Suppl. Mat. 12) caused the destruction of fruit that was already growing on the plants. Flooded farmers reported on average 83% of ongoing production was destroyed. The majority (77%) of banana plants in flooded areas were destroyed during the inundation with water and subsequent submersion period. Observations from surveys are also reflected in our regional-scale remote sensing analyses, where the canopy signature of flooded banana plantation pixels showed a sharp deviation away from values for non-flooded banana plantation pixels immediately after the hurricanes (Fig. 3(a)), indicating a rapid change in the canopy structure of flooded plantations. Based on the PFI—a surrogate for the proportion of productive banana plantation pixels—we observed losses of production area continuing until mid-December 2017 (Fig. 3(b)) before signs of production capacity recovery were detectable. This suggests that the true extent of production area loss is not immediately apparent after the initial hurricane shock, but accumulated up to 3 months after the event. Beyond the damage to banana plants, there was also significant infrastructure damage, with 18% of farmers experiencing cable ways being destroyed, 15% with packhouse damage, 68% with drainage canals destroyed and 71% having roads on their farms destroyed (Suppl. Mat. 12).

### Recovery time is highly variable between farmers

After flooding, there are a key set of activities that farmers reported performing to return banana production to full capacity (Suppl. Mat. 13). These are moderated by natural processes after the flood event, such as the drainage of flood waters and soil aeration. Following this, the farmers and labourers cultivated the field using traction, prepared drains and paths and planted seed material. After the replanting phase, there was a 9-month growth phase before fruits were harvestable and saleable. Concurrently, importers’ and exporters’ recovery process involved assessing losses from existing contracted farmers and then switching their sourcing to other locations not hit by the hurricane within the DR, as well as abroad. Coordination with other suppliers to fill gaps in order fulfilment was also performed by importers and exporters. The government response involved repairing damage to major infrastructure, as well as the provision of financial support to farmers and the purchasing of fruit from farmers that had lost market access.

**Fig. 4:**
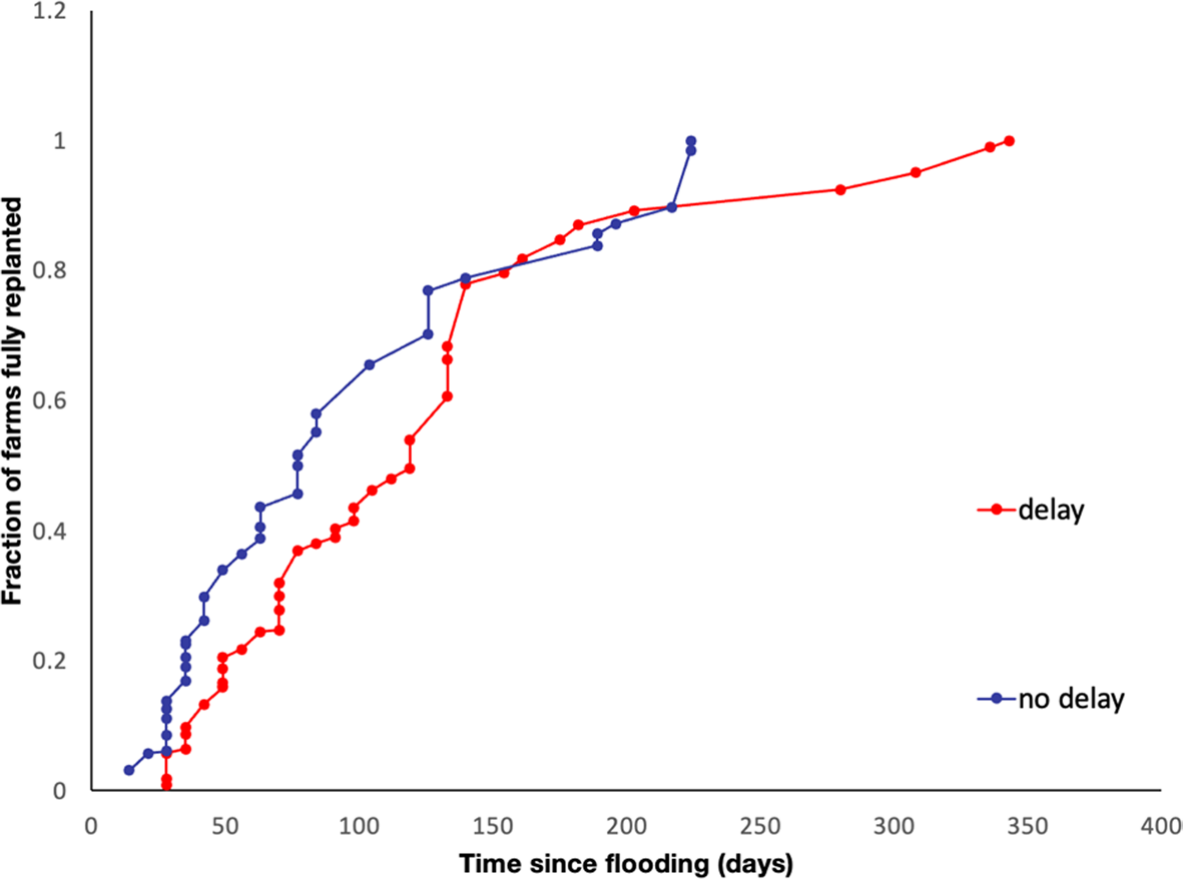
Agricultural recovery trajectories of flooded banana farmers (*n* = 80) based on recall of key events in 2017 and 2018 after hurricane-induced flooding. Farmers with delays in to starting replanting (red) versus farmers without delays (blue). The *x* axis represents time since inundation in days and the *y* axis represents the cumulative fraction of the banana production area that is planted. Each point represents the completion of replanting for one farmer in the sample. The time difference between the completion of replanting and productive recovery assessed by remote sensing (Fig. 3) is related to the time after replanting that banana canopies have regrown

At the farm scale, we found large variation in recovery times between farmers (Fig. 4). Recovery times, reported by farmers and considered from an agricultural perspective (marketing aspects are covered in Sect. 2.4), covering the time between fields draining and completion of replanting, ranged from 2 weeks to more than 11 months (min = 14 days, max = 343 days, mean = 99 days), with the difference between the slowest quartile and fastest quartile of recovery times being 91 days. The dynamics of the recovery process were significantly affected by delays (Fig. 4), the time between when farmers judged fields were ready to cultivate and when they were effectively able to start. Fifty-three percent of surveyed farmers that were flooded (42 farmers) experienced delays in replanting. These delays vary between 7 and 352 days, with a mean delay of 35 days. For farmers that experience delays, it on average increased the overall recovery time by 96% and therefore significantly inhibited the recovery process.

Our analyses of farmer recovery data (Suppl. Mat. 6–8) found four factors that influence smallholder recovery times (Table 1): (1) *Scale of damage* (based on flooded area, replanted area, total banana farm size; β= 0.262, *t* = 2.12, *p* < 0.05), (2) *farm and livelihood diversity* (agricultural crop diversity, non-agricultural income diversity, banana income dependency; β= 0.240, *t* = 2.21, *p* < 0.05) and (3) *drainage time* (β = 0.298, *t* = 2.99, *p* < 0.01) all increased recovery time. In contrast, (4) *farmer flood training* (flood protection training, flood recovery training; β = − 0.256, *t* = − 2.65, *p* < 0.01) made recovery quicker. These findings were supported and augmented by qualitative evidence from interviews, with cooperatives and farmers citing flood water drainage, availability of finance to purchase materials and labour for cultivation and replanting, as well as the availability of planting material as major constraints to recovery. We found farmers were supported in several ways, including by the cooperatives, who reported having to take out bank loans using their office buildings as collateral.

**Table 1:** Key factors effecting smallholder recovery time post-hurricane-induced flooding. This presents the standardised coefficient estimates from the multiple linear regression where the response variable is recovery time. Variables with negative coefficients speed up recovery, whilst those with positive values slow down recovery (significance: *p* < 0.01 ‘**’, *p* < 0.05 ‘*’). The model has an adjusted R squared value of 0.241, showing it explains 24% of the variation in recovery time. Recovery time = β_0_ + β_1_ Scale of damage + β_2_ Farm and livelihood diversity + β_3_ Flood training + β_4_ Drainage + ε

Variables	Standardised regression coefficients	Standard error
(Intercept)	1.233**	**0.0997**
Scale of damage	0.262*	**0.0999**
Farm and livelihood diversity	0.240*	**0.0998**
Flood training	− 0.256*	**0.0998**
Drainage	0.298**	**0.1000**
Multiple R-squared: 0.2807,Adjusted R-squared: 0.241		
F-statistic: 7.026 on 4 and 72 DF. *p* < 0.0001		

Whilst farmer surveys capture recovery in terms of the time required to prepare and then replant farms, remote sensing analyses gave us a clearer picture with respect to trajectory of recovery of regional productive capacity. Based on banana canopy backscatter values from SAR data, and using lenient criteria to define recovery, we found that production recovery (replanting and regrowth) was completed, at the earliest, by June 2018 (Fig. 3(a)). However, canopy signatures of flooded plantations began tracking that of non-flooded plantations more closely only by late September 2018—approximately 380 days after the first hurricane (Fig. 3(b)). Applying more stringent criteria using the PFI, we estimate that the region’s productive capacity returned to pre-hurricane levels by the beginning of December 2018, approximately 450 days after the first hurricane (Fig. 3(b)). Hence, DR’s banana production system is likely to have seen below capacity production for a period of 15 months due to hurricanes Irma and Maria. Remote sensing analyses also revealed that by 2019, production area had exceeded pre-hurricane area under cultivation by 10.8% (loss of area = 5048 ha; gain of area = 7385 ha; net gain = 2337 ha). However, 26.9% of new plantation area co-occurred at locations that had experienced damage from hurricanes Irma and Maria. This represents a net increase of 2.9% of plantation area at risk from the reoccurrence of a similar extreme weather event (Suppl. Mat 14).

### Exposure to global markets leads to spillover of flooding impacts beyond those experiencing direct damage

**Fig. 5:**
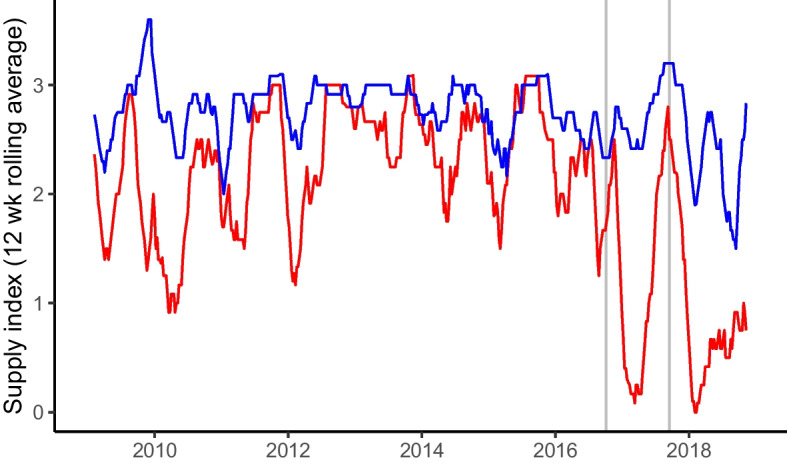
UK banana wholesale imports from the Dominican Republic from 2009 to 2019. The red line shows Dominican Republic banana wholesale imports to the UK represented as a supply index (0 = unavailable, 1 = scarce: 5 = glut, aggregated as a 12-week rolling average. The blue line shows Colombian banana imports to the UK for comparison. The two vertical grey lines represent Hurricane Matthew (hit DR on 4th Oct 2016) and Maria (16th September 2017)

Recovery is determined by both farmers replanting their crop, and the ability to sell their produce and thus generate income to replenish household and farm assets. This in turn is influenced by responses of the downstream GFVC. Interviewed multi-national importers reported switching sourcing to other countries, e.g. Mexico—an emerging region for organic banana production—in the aftermath of the hurricanes. Consequently, 43% of surveyed farmers in the DR reported market inaccessibility in the following year, which impacted both flooded (40% of surveyed farmers) and non-flooded (45%) farmers. However, on average, flooded farmers saw greater reductions in the proportion of production sold to the export market (30% of harvested production) compared to non-flooded farmers (21%). These survey data are supported by flooded farmers reporting a reduction in income and cash flow in interviews and during the workshop. Additionally, these farm-scale results are supported by downstream UK import data which show a rapid decrease in imports from the DR following the hurricane events in 2017 and a limited recovery in the subsequent months (Fig. 5).

## Discussion

Globally, the food system is under increasing threat from disruptions caused by extreme weather events, such as hurricanes and droughts. The scarcity of information about how these shocks impact the interconnected actors in these systems, particularly vulnerable smallholder farmers, is a barrier to enhancing the resilience of the food system. Here we have shown that the flood damage caused by hurricanes Irma and Maria to the banana production system in the Dominican Republic was substantial. Additionally, the process of recovering to pre-hurricane production capacity was protracted, taking up to 15 months, with consequences for farmers’ cashflow, overall income and national exports. The smallholder farmers had very limited power to mitigate hurricane-induced flood damage, particularly beyond the farm scale. Consequently, the shock had an ‘all-or-nothing’ nature, with exposed farmers experiencing complete destruction of their banana farms. In the face of such intense damage, we find recovery becomes a critical phase in determining the system’s resilience. Our analyses show that increased farm and livelihood specialisation, as well as flood training, quickened recovery and that increased scale of flood damage and drainage time slowed recovery. Beyond this, by engaging in a GFVC, farm recovery was impacted by a ‘double exposure’ from production and market loss, the latter creating a ‘spillover’, affecting both flooded and non-flooded farmers alike. Hence, even though our remote sensing analyses directly observed 11% of regional production area affected by flooding, the spillover effect of being part of a GFVC increased the proportion of DR banana producers impacted by this shock. These findings have several implications for the understanding of resilience in food systems, the design of supply chain initiatives to support smallholder farmers and future food systems research, which we enumerate in our conclusion.

We show that the GFVC actors’ recovery actions influence outcomes from extreme weather at multiple scales, including at a farm, cooperative and national scale. This is an issue in food system sustainability interventions that has been underprioritised historically, with more interventions and research focusing on robustness to hazards such as, for example, developing drought-tolerant varieties. This is particularly problematic as many aspects of climate change are already ‘baked in’; i.e. its effects are unavoidable, already being felt and frequently catastrophic. In this context, a narrow focus on only developing strategies to enhance robustness of food systems to these threats is unlikely to keep pace with the impacts already being felt. The lack of adequate recovery strategies will only exacerbate the impacts of climate change, especially for vulnerable groups (e.g. smallholder farmers). The COVID-19 crisis has drawn more attention to recovery from shocks in food systems (Barman et al. 2021), supporting our finding of the key importance of recovery speed and quality (e.g. recovery strategies that also put in place measures to protect against future shocks). Beyond this, the recovery phase can also present an opportunity for GFVC actors to adapt or even transform their systems, with our study suggesting mechanisms (e.g. landscape collaboration and spatial targeting of training—discussed as follows) by which this could be delivered.

Our finding that there is high heterogeneity in farmer recovery times demonstrates this phase presents an opportunity to enhance smallholder resilience and to level up these differences between farmers. The speed of recovery affects farm productivity (Perfecto et al. 2019), as well as food security outcomes (Rakotobe et al. 2016), and becomes increasingly important when shocks are of a high frequency where there is limited time to replenish assets. These farm-scale recovery trajectories have national economic consequences, for countries that are highly dependent on agricultural commodity production, with slower recoveries costing economies hundreds of millions of dollars (Honduras banana exports recover from hurricane damages – Produce Blue Book n.d). These findings highlight the need for further research into ways that recovery rates can be increased after extreme weather events.

Previous research has suggested that increased diversity enhances farmer’s resilience to climate shocks (Aguilar-Støen et al. 2009; Melvani et al. 2020; Abson et al. 2013). However, our findings suggest the contrary for banana farmers in the DR. In follow-up interviews and focus groups, farmers identified certification requirements as their key motivation to diversify agricultural production, and that income was limited from secondary crops. This highlighted the challenge of having ‘functional diversity’ that can provide additional benefits to a livelihood strategy, such as additional income or nutrition. We conclude that whilst diversification is encouraged and to some extents implemented (e.g. intercrops and boundary crops), they do not play a significant functional role in the famers’ livelihoods and, consequently, do not enhance farmer resilience.

The severity of the hurricanes meant that farmers were unable to prevent damage to their farms, resulting in the ‘all-or-nothing’ nature of damage that stands in sharp contrast to more incremental impacts of other types of extreme events (e.g. droughts (Harvey et al. 2018)). This suggests preventative measures require actions beyond individual farms, and collaborative efforts between farmers and cooperatives at appropriate scales (e.g. the drainage basin). Our transdisciplinary approach elicited measures that are viewed as desirable by local stakeholders, including landscape management to increase forestation in drainage basins, as well participatory planning with government, water boards and farmers to optimise zoning of agricultural land and the location of flood defences. Supply chain sustainability interventions could play a valuable role in facilitating the necessary collaborations. However, we did find some evidence of beyond farm scale coordination, in the form of cooperatives acting as communication hubs and also risk pools between farmers. This extends the claim that cooperatives can play a key role in enhancing smallholder farmers’ livelihoods (Bacon et al. 2014), in this case via contributions to climate resilience.

Topographical elements that drive flood risk exposure, captured by factors ‘scale of damage’ and ‘drainage’, highlight the potential of risk-based spatial planning in land-use decision-making in flood-prone areas. This suggests that production sites that are both flood prone and difficult to drain should be avoided, when possible, as suggested by Philpott et al. (Philpott et al. 2008). Our satellite image analysis in the DR shows that between 2017 and 2019, 26.9% of newly added plantation area in the region were in locations that experienced hurricane damage in 2017. Consequently, the overall plantation area under risk from a similar extreme event increased by 2.9%. Hence, whilst the scale of damage is often out of the control of the farmer, the site choice for banana production (or switching to alternative crops) can be better informed if flood risk and drainage potential are taken into account, from both a damage and recovery perspective.

Our finding that training farmers in specific flood damage prevention and flood recovery strategies is influential in reducing their recovery time highlights a key mechanism that has been used widely to improve farmer agronomic strategies but not widely within resilience enhancement strategies IISD 2013. Given that risk exposure is clearly identifiable based on distance to river (Suppl. Mat. 5) and previous hurricane events, expanding training to all farmers in ‘risk zones’ could enhance both individual recovery outcomes and regional-scale economic responses post-event.

Our results show that farmers in a global food GFVC experiencing a flood shock face a double exposure, i.e. weather-driven production loss and simultaneous market access loss. This provides evidence to support the notion that climate change and globalisation will act together to disrupt vulnerable populations (O’Brien and Leichenko 2000). Market exposure increases the scope of those that are impacted by the shock to non-flooded farmers. Even though 95% of sampled farmers are Fairtrade certified, market responses after a production shock are not fully covered by the scope of existing Fairtrade farmer-buyer agreements and results in buyers abandoning farmers at their most vulnerable moment. This provides further evidence for the need to enhance long-term relationships between farmers and buyers (Ola and Menapace 2020).

## Limitations

The overall transdisciplinary approach utilising mixed methods, comprising remote sensing, participatory activities and a household survey, allowed us to capture a holistic picture of how extreme weather events affect smallholder farmers engaged in GFVCs. These methods had some limitations, and we highlight two here. A key limitation of this study, and potential avenue for future research, was the scale at which damage and recovery was assessed. We chose to assess at two spatial scales, at the regional scale (in the key producing regions) and at the farm (and household) scale. However, it is clear that impacts of the hurricanes were felt at other scales, such as the community (e.g. village infrastructure, loss of employment) and at the landscape scale (e.g. off-farm environmental damage). These two scales would provide additional context to how extreme weather events impact smallholder farmers. Beyond this, the temporal scale at which we assessed recovery, up to 24 months after the shocks occurred, allowed us to assess agricultural and marketing impacts. However, it has been shown from other studies of extreme weather events (e.g. Dercon (Dercon 2004)) that impacts on household consumption, for example, can be seen even 15 years later. Therefore, it would be valuable to conduct follow-up studies using panel data on additional aspects of recovery, such as household consumption.

## Conclusion

Our findings have several implications for the design of supply chain initiatives to support smallholder farmers and future food system resilience research. Firstly, to enable smallholders to reduce their exposure to such hazards, new mechanisms to promote collaborations between farmers and or cooperatives beyond the farm scale (for example, at a drainage basin scale) should be explored. Secondly, our findings show that recovery interventions should be expanded, in a risk-targeted way, to more farmers by GFVC actors, such as certifiers and traders, as it is underprioritised in existing initiatives. Thirdly, we demonstrate the need for initiatives to be designed in a way that allow farmers engaged in GFVCs to maintain market access after production shocks, so as to avoid a ‘double exposure’ and its associated spillovers to farmers initially unaffected by the shock. Overall, this study reveals the high interdependence of actors in smallholder GFVCs with regard to resilience to extreme weather events and signals that mechanisms to promote solidarity between actors and the adoption of responsible resilience strategies should be sought to protect against the negative impacts of such events.

## Supplementary Information

Below is the link to the electronic supplementary material.

Supplementary file1 (DOCX 10533 KB)

## Data Availability

The datasets generated during and/or analysed during the current study are not currently publicly available due to data privacy obligations under grant MRP16OrRes but are available from the corresponding author on reasonable request.
