# Supplementary material for: Smallholder farmer resilience to extreme weather events in a global food value chain
Source: Clim Change. 2023 Oct 30;176(11):152. doi: 10.1007/s10584-023-03586-1 (PMC11779771; doi:10.1007/s10584-023-03586-1)
Supplement: Supplementary file 1 — Supplementary file1 (DOCX 10533 KB) [file 10584_2023_3586_MOESM1_ESM.docx]

**Supplementary Material: Smallholder Farmer Resilience to Extreme Weather Events in Global Food Value Chains**

**SM1: Methodology flowchart** The flowchart gives an overview of how the various methods interact (grey arrows indicating interactions).

**
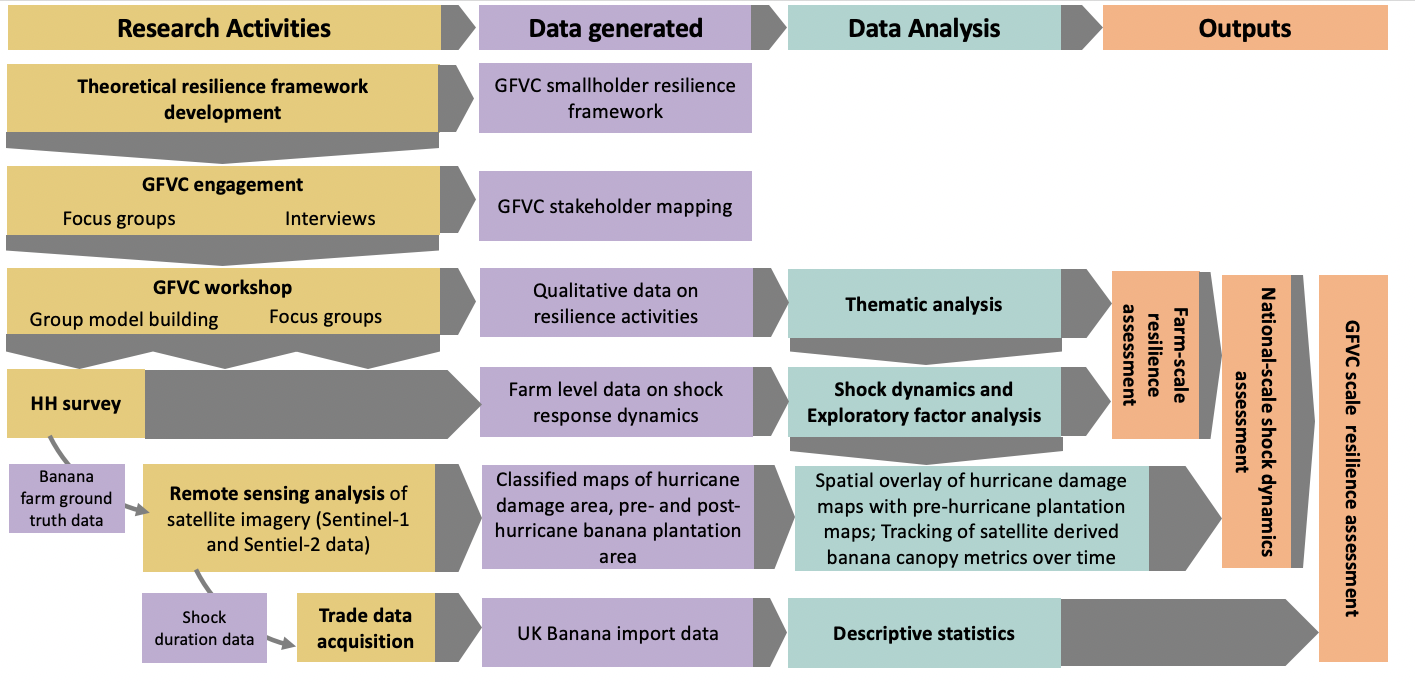
**

**SM2: Overview of workshop participants** The following actors were involved in the study (ranging from workshop participation to interviews and data sharing): national and regional government, NGOs, DR regional waterboard, sustainability certifiers, farmer cooperatives, smallholder farmers, large-scale producers, DR exporters, multinational importers and a UK retailer.

**
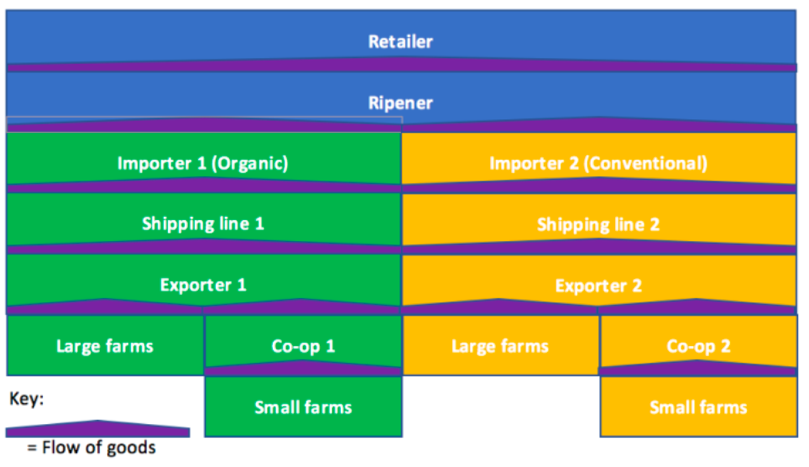
**

**SM3: UK trade data acquisition** UK banana trade data (for Birmingham and New Spitalfields wholesale markets) were obtained from https://www.gov.uk/government/statistical-data-sets/banana-prices. Supply categories were 0 no supply, 1 scarce, 2 small, 3 moderate/ample, 4 heavy and 5 glut. Running means (12 week) were used for visualization.

**SM4: HH survey farmer sampling:** We did this using farmer GPS location data shared by farmer cooperatives. Using a zone generated from a rivers layer on GIS software, we included farms within 1.5 km of a waterway to make up 60% of the sample^55^. Using the lists generated from this analysis of farmer cooperative data, we randomly sampled farmers from the list of farmers in the buffer zone and a list of farmers whose farms are outside the buffer zone (40%).

**SM5:** **Characteristics of sampled banana farmers** Means are presented with standard deviation in brackets for continuous and count variables. Differences are assessed using t-tests for continuous variables and Chi-squared for binary and count). Farmers were categorised based on whether they were flooded as a result of the 2017 hurricanes.

| **Characteristic** | **Total sample, n = 158** | **Non-flooded, n = 78** | | **Flooded in 2017 n = 80** | | **P value** | |  |
| --- | --- | --- | --- | --- | --- | --- | --- | --- |
| Female | 11% | | 8.10% | | 15% | | 0.248 | |
| Age | 50 (13) | | 50 (14) | | 51 (12) | | 0.286 | |
| Number of farms | 1 (1) | | 1 (1) | | 1 (1) | | 0.072 | |
| Farm size (ha) | 5.2 (4.8) | | 5.1 (5.2) | | 5.2 (4.3) | | 0.176 | |
| Organic | 78% | | 74% | | 83% | | 0.245 | |
| Fairtrade | 95% | | 94% | | 96% | | 0.915 | |
| RA | 47% | | 48% | | 46% | | 0.943 | |
| Intercrops | 1 (1) | | 1 (1) | | 1 (1) | | 0.822 | |
| Crop types | 2 (2) | | 2 (1) | | 2 (2) | | 0.725 | |
| Non ag. income diversity | 0 (1) | | 0 (1) | | 0 (1) | | 0.600 | |
| **Distance to river (m)** | **1698 (2263)** | | **2232 (2570)** | | **1164 (1772)** | | **0.007** | |
| Banana income proportion | 0.61 (0.31) | | 0.61 (0.31) | | 0.62 (0.32) | | 0.821 | |

**SM6:** **Variables hypothesised to determine farmers recovery from flooding event:**

| **Name** | **Description** | **Expected influence on recovery time (RT)** | **Literature supporting variables role in climate resilience of smallholders** |
| --- | --- | --- | --- |
| Gender | Female household head | Positive (i.e increased RT) | (Jost et al. 2016) |
| Age | Age of household head | Negative | (Tazeze, Haji, and Ketema 2012) |
| Education | Number of years education | Negative | (Menike and Arachchi 2016) |
| Banana income dependency | Proportion of income form bananas | Positive |  |
| Non-agricultural income streams | Number of non-agricultural income sources | Negative | (Bellon et al. 2020) |
| Diversity of all income streams | Number of different income sources (ag./non-ag.) | Negative | (Antwi-Agyei, Stringer, and Dougill 2014) |
| Farmer owned | Household owns the farm | Negative | (Antwi-Agyei, Dougill, and Stringer 2015) |
| Banana farm size | Area of banana farms in hectares | Negative | (Harvey et al. 2014) |
| Distance from river | Distance from the river in metres | Negative | (Philpott et al. 2008) |
| Intercrops | Number of different crops integrated in banana production | Negative | (Lasco, Delfino, and Espaldon 2014) |
| Farm diversity | Diversity of crops produced on the farm | Negative | (Lin 2011) |
| Flooded area | Size of area flooded in September 2017 | Positive |  |
| Area replanted | Area of banana farm that was replanted after flooding | Positive |  |
| Agricultural network size | Number of other farmers that the farmer discusses banana production strategy with every month | Negative | (Saint Ville et al. 2016) |
| Flood training | Farmer has received specific training on flood damage prevention | Negative | (Nor Diana et al. 2019) |
| Recovery training | Farmer has received specific training on replanting the farm after flooding | Negative | (Stewart et al. 2015) |
| Agricultural group memberships | No. of agricultural groups the farmer is engaged in | Negative | (Kangogo, Dentoni, and Bijman 2020) |
| Certification | Farm is certified under Organic, RA or FT | Negative |  |
| Financial sources | Number of financial sources the farmer has access to e.g. bank loans, credit groups | Negative | (Li et al. 2020) |
| Savings | Does the farmer have savings | Negative | (Oostendorp et al. 2019) |
| Insurance | Does the farmer have flood insurance | Negative | (Collier, Skees, and Barnett 2009) |
| Drainage time | Days after initial flood water drained from farm | Positive |  |

**SM7: Correlation matrix of the explanatory and dependant variables.** Only significant correlations (p<0.05) are displayed. Circle size and colour intensity are proportional to the correlation coefficients (Pearson's) Blue circles indicate positive correlation and red circles indicate negative correlation. Recovery time (dependent variable) and its sub-components are on the right-hand side of the matrix. Income diversity was replaced by non-agricultural diversity (income diversity – ag. diversity) as its correlation with ag. diversity was above 0.9. These nine variables met the KMO measure of sampling adequacy to be included in the factor analysis (>=0.5, SM2). The overall KMO with these nine variables was 0.58. A Bartlett’s test of sphericity on these variables (Chi squared = 329.97, p<0.01) indicated that there was sufficient correlation between the variables to conduct a factor analysis.


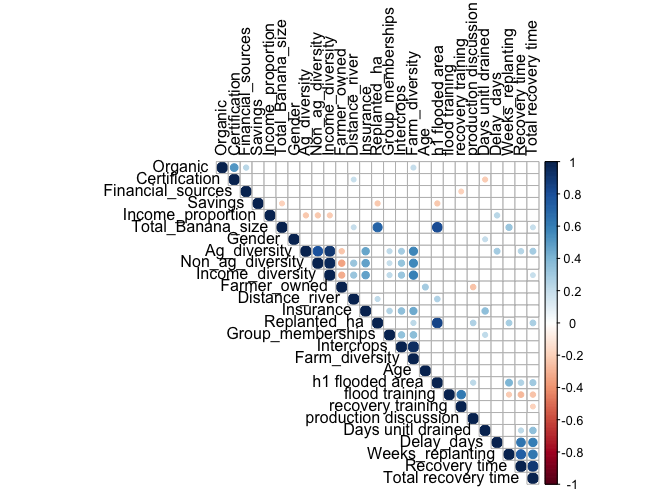


**SM8: Factor loadings after rotation.** Loadings greater than 0.3 are considered in the interpretation of the factors and highlighted in bold.

|  | **Scale of damage** | | **Farm and livelihood diversity** | **Flood Training** | **Drainage** |
| --- | --- | --- | --- | --- | --- |
| Flooded area | | **0.94** | 0.01 | -0.01 | 0.10 |
| Replanted area | | **0.87** | 0.13 | -0.01 | -0.05 |
| Total banana farm size | | **0.91** | -0.05 | -0.03 | 0.11 |
| Agricultural crop diversity | | 0.05 | **0.93** | 0.09 | 0.04 |
| Non. ag. income diversity | | 0.02 | **0.92** | -0.05 | -0.03 |
| Flood training | | -0.08 | -0.02 | **0.90** | -0.10 |
| Recovery training | | 0.04 | 0.05 | **0.88** | 0.18 |
| Drainage time | | 0.14 | 0.19 | 0.01 | **0.82** |
| Banana income dependency | | -0.03 | **-0.40** | 0.08 | **0.57** |
| Eigen values | 2.49 | | 1.93 | 1.60 | 1.07 |

**SM9: Mapping banana production area and impact of hurricanes**

In brief, banana plantations for 2019 were mapped at a resolution of 10 m x 10 m by building a random forest classifier using a fusion of Synthetic Aperture Radar (SAR) data from the European Space Agency (ESA) Sentinel-1 satellite platform, multi-spectral data from ESA’s Sentinel-2 platform, and terrain information from the 90m resolution Shuttle Radar Telemetry Mission (SRTM) Digital Elevation Model (DEM). Specifically, classification was run on a stack of rasters comprising the median VV polarisation backscatter for 2019 (i.e. January 2019 to December 2019), the standard deviation in VV backscatter for the same time period, the median red, green, blue and NDVI values for 2019, and slope derived from the DEM. The random forest classifier was trained using ground truth data from 100 banana plantation polygons, and a set of other land-cover classes that were manually digitised using imagery available from Google Earth. The classifier was used to produce a map of banana plantation area for 2019 (representing the post-hurricane recovered production area). Using a confusion matrix^6^^0^ and a random sample of 500 test pixels generated from the banana plantation ground truth data, accuracy of classified banana plantations was estimated at 99.8%. The trained classifier was then used to produce a map for pre-hurricane banana plantations using one year’s worth of Sentinel-1 and Sentinel-2 data immediately preceding hurricane Irma (i.e. from September 2016 to August 2017).

The area affected by the hurricanes was mapped using ESA’s Sentinel-1 data. Affected areas consisted of the spatial union of three components. First, the immediate impact of the hurricanes was identified as pixels with large reductions in VV polarisation backscatter immediately after the hurricane events, as reduced backscatter can be used as a signal for flooded pixels^6^^1^. The second component comprised a 100 meter buffer around the pixels identified in the first component. This accounted for areas that are likely to have experienced open water flooding (or at the very least, inundated soils) but which may have been obscured by vegetation features (e.g. banana plants, large trees, etc.). The third component, a legacy effect, was identified as pixels which during the three months after the hurricane events showed large negative deviations in average VV polarisation backscatter values relative to the distribution of values observed for the year preceding the hurricanes. This third component accounts for more protracted impacts of the hurricane, for example, banana plantation pixels that do not see immediate loss of plants after the hurricane. Spatially overlaying the pre-hurricane banana plantation area map (for 2017), the post-hurricane plantation map (for 2019) and the map of areas affected by hurricanes in the region, we identified (1) the location and spatial extent of banana plantation area in 2017 affected by the hurricane events, (2) the turnover in plantation area between 2017 and 2019, and (3) plantations area in 2019 that would be at risk if a similar extreme weather event were to occur again. A detailed description of the remote sensing methods can be found in Varma et al.^5^^9^.

**SM10: Quantifying recovery of banana plantations**

A set of 6500 random sampling points were generated within each group, such that minimum spacing between points was 50m. Sentinel-1 VV polarisation backscatter values, averaged within a 50m x 50m window, were extracted at each sampling point from every Sentinel-1 image available from March 2017 to April 2018 (68 images). The spatial averaging in a 50m window was conducted to eliminate speckling artefacts that SAR data suffers from when working at fine spatial resolutions. Separately for the flooded and non-flooded pixels, we calculated the first quartile (Q1), median (Q2) and third quartile (Q3) of the VV backscatter values across the study region for every date that Sentinel-1 data were available for. These data (i.e. Q1, Q2 and Q3) were visualised as a function of date of image capture to illustrate the deviation in backscatter values in flooded pixels after the hurricane events relative to non-flooded pixels.

**SM11: Proportion of farmers utilising different strategies to enhance their resilience to a flooding event.** Flooded farmers (green) and non-flooded farmers (blue) are segregated, with significant differences (Chi-squared test) shown (p<0.01= **).


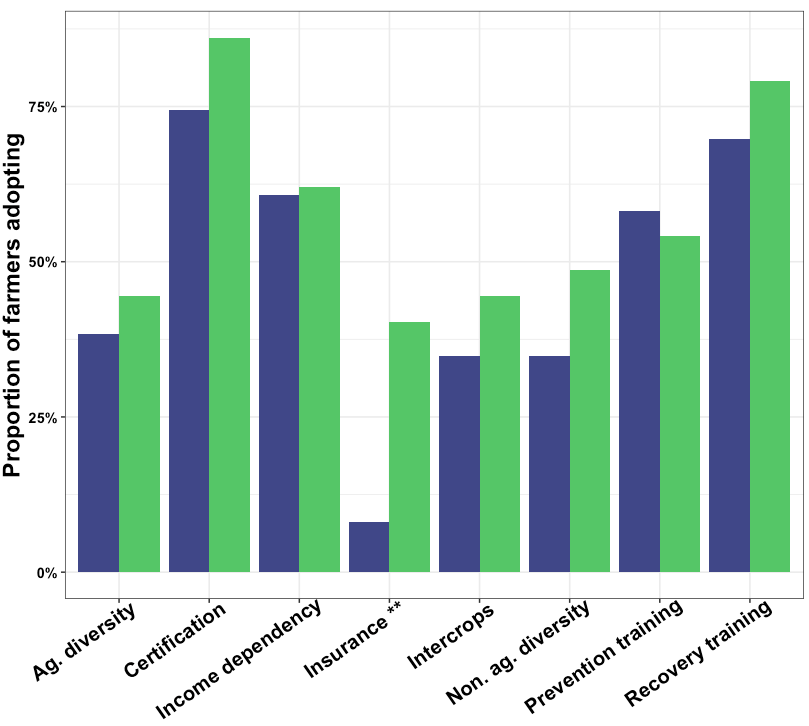


**Flooded farmers**

**Non-Flooded farmers**

**SM12: a)** Fraction of flooded farmers experiencing different types of damage (infrastructure is proportion of farmers reporting incident, production is mean proportion of land of production destroyed. **b)** Extent of flood waters on banana farm in Valverde province 2017. **c)** Damage to banana plants after flood water receded


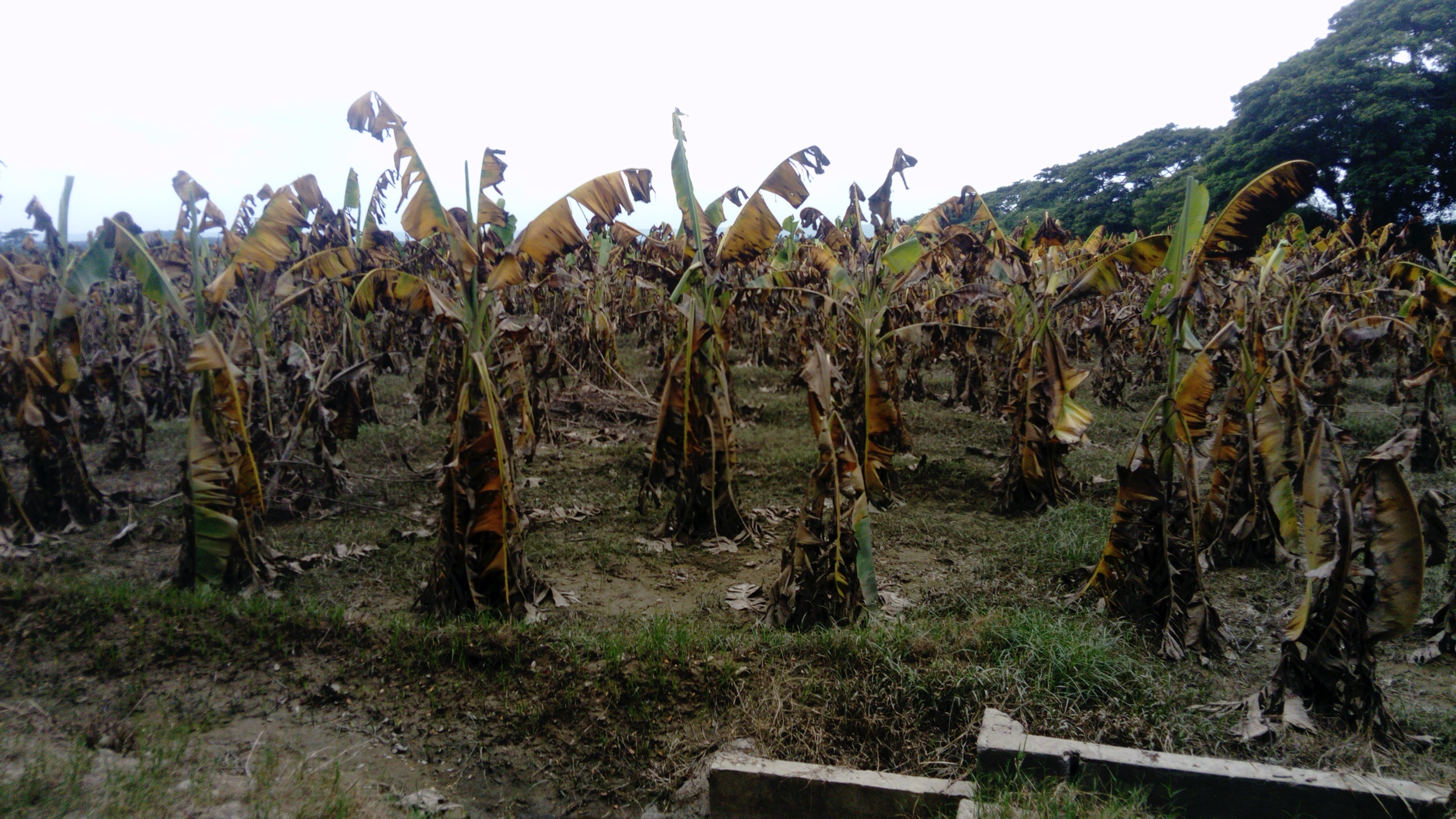

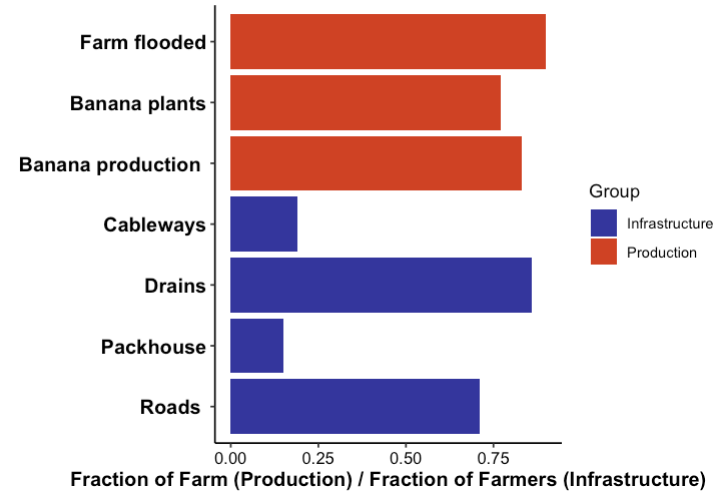


**a) Damage**


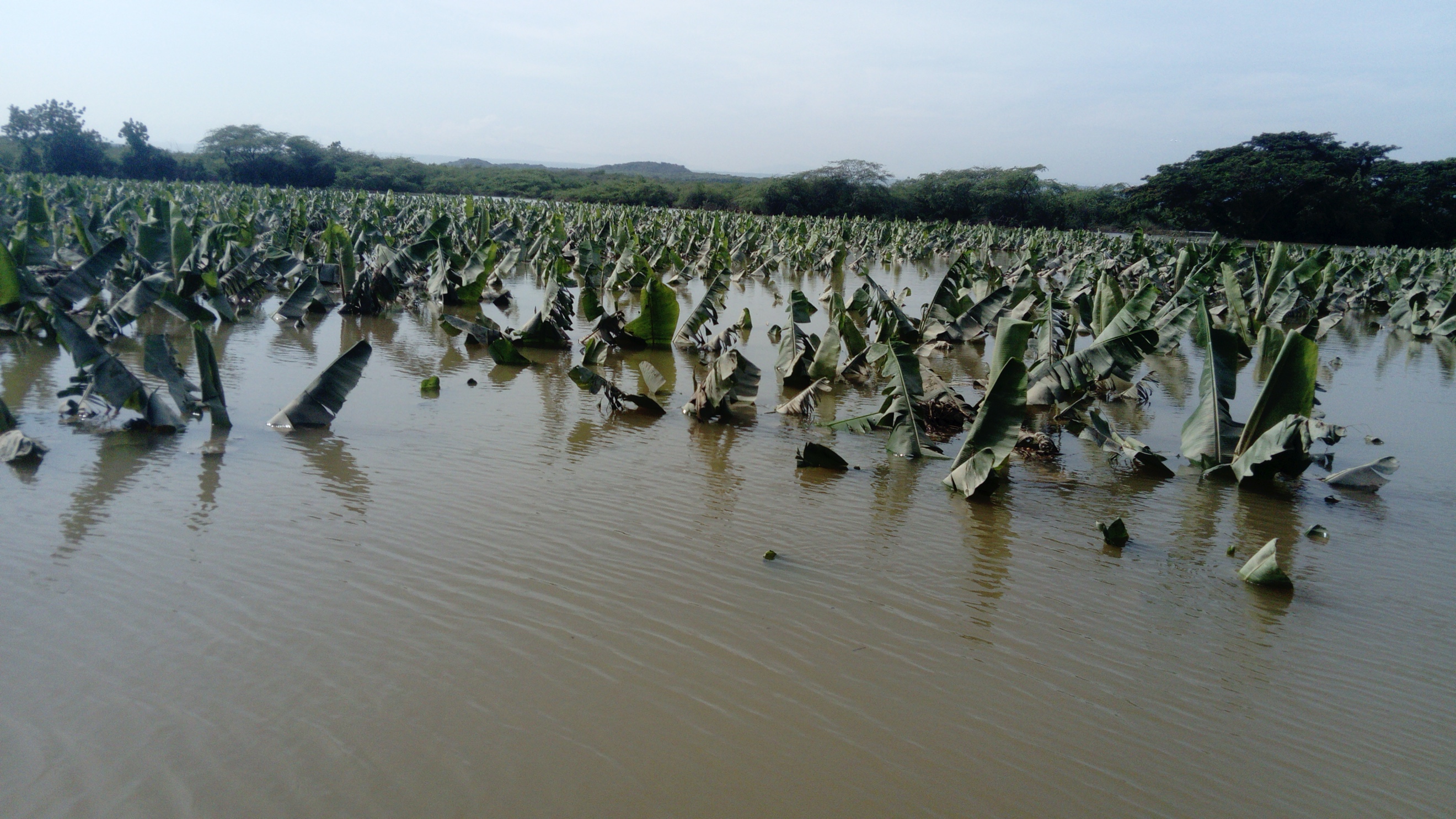


**b) Flood waters in 2017**

**c) Damage to banana plants**

**SM13: Agricultural recovery process for smallholder banana farmers after flooding**


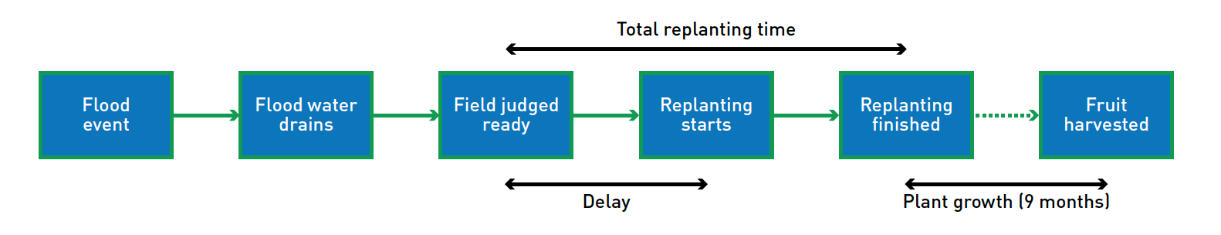


**SM14: Banana plantation areas in 2019 that overlap with locations that saw storm-related damage due to hurricanes Irma and Maria in 2017.** The highlighted regions (red) are considered at high risk should similar extreme weather events reoccur.

**
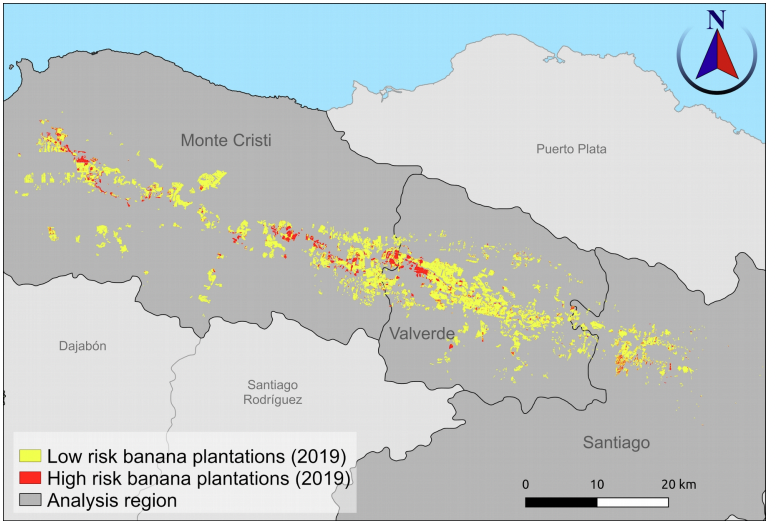
**

**SM References:**

Antwi-Agyei, Philip, Andrew J. Dougill, and Lindsay C. Stringer. 2015. “Impacts of Land Tenure Arrangements on the Adaptive Capacity of Marginalized Groups: The Case of Ghana’s Ejura Sekyedumase and Bongo Districts.” *Land Use Policy* 49 (December): 203–12. https://doi.org/10.1016/j.landusepol.2015.08.007.

Antwi-Agyei, Philip, Lindsay C. Stringer, and Andrew J. Dougill. 2014. “Livelihood Adaptations to Climate Variability: Insights from Farming Households in Ghana.” *Regional Environmental Change* 14 (4): 1615–26. https://doi.org/10.1007/s10113-014-0597-9.

Bellon, Mauricio R., Bekele Hundie Kotu, Carlo Azzarri, and Francesco Caracciolo. 2020. “To Diversify or Not to Diversify, That Is the Question. Pursuing Agricultural Development for Smallholder Farmers in Marginal Areas of Ghana.” *World Development* 125 (January): 104682. https://doi.org/10.1016/j.worlddev.2019.104682.

Collier, Benjamin, Jerry Skees, and Barry Barnett. 2009. “Weather Index Insurance and Climate Change: Opportunities and Challenges in Lower Income Countries.” *Geneva Papers on Risk and Insurance: Issues and Practice* 34 (3): 401–24. https://doi.org/10.1057/gpp.2009.11.

Harvey, Celia A., Zo Lalaina Rakotobe, Nalini S. Rao, Radhika Dave, Hery Razafimahatratra, Rivo Hasinandrianina Rabarijohn, Haingo Rajaofara, and James L. MacKinnon. 2014. “Extreme Vulnerability of Smallholder Farmers to Agricultural Risks and Climate Change in Madagascar.” *Philosophical Transactions of the Royal Society B: Biological Sciences* 369 (1639): 20130089. https://doi.org/10.1098/rstb.2013.0089.

Jost, Christine, Florence Kyazze, Jesse Naab, Sharmind Neelormi, James Kinyangi, Robert Zougmore, Pramod Aggarwal, et al. 2016. “Understanding Gender Dimensions of Agriculture and Climate Change in Smallholder Farming Communities.” *Climate and Development* 8 (2): 133–44. https://doi.org/10.1080/17565529.2015.1050978.

Kangogo, Daniel, Domenico Dentoni, and Jos Bijman. 2020. “Determinants of Farm Resilience to Climate Change: The Role of Farmer Entrepreneurship and Value Chain Collaborations.” *Sustainability* 12 (3): 868. https://doi.org/10.3390/su12030868.

Lasco, Rodel D., Rafaela Jane P. Delfino, and Marya Laya O. Espaldon. 2014. “Agroforestry Systems: Helping Smallholders Adapt to Climate Risks While Mitigating Climate Change.” *Wiley Interdisciplinary Reviews: Climate Change* 5 (6): 825–33. https://doi.org/10.1002/wcc.301.

Li, Wenjing, Chuanmin Shuai, Yu Shuai, Xin Cheng, Yue Liu, and Fubin Huang. 2020. “How Livelihood Assets Contribute to Sustainable Development of Smallholder Farmers.” *Journal of International Development* 32 (3): 408–29. https://doi.org/10.1002/jid.3461.

Lin, Brenda B. 2011. “Articles Www.” *BioScience* 61 (3). https://doi.org/10.1525/bio.2011.61.3.4.

Menike, L.M.C.S., and K.A.G.P. Keeragala Arachchi. 2016. “Adaptation to Climate Change by Smallholder Farmers in Rural Communities: Evidence from Sri Lanka.” *Procedia Food Science* 6 (January): 288–92. https://doi.org/10.1016/j.profoo.2016.02.057.

Nor Diana, M. I., S. Chamburi, T. Mohd Raihan, and A. Nurul Ashikin. 2019. “Assessing Local Vulnerability to Climate Change by Using Livelihood Vulnerability Index: Case Study in Pahang Region, Malaysia.” In *IOP Conference Series: Materials Science and Engineering*, 506:012059. Institute of Physics Publishing. https://doi.org/10.1088/1757-899X/506/1/012059.

Oostendorp, Remco, Marcel van Asseldonk, John Gathiaka, Richard Mulwa, Maren Radeny, John Recha, Cor Wattel, and Lia van Wesenbeeck. 2019. “Inclusive Agribusiness under Climate Change: A Brief Review of the Role of Finance.” *Current Opinion in Environmental Sustainability*. Elsevier B.V. https://doi.org/10.1016/j.cosust.2019.09.014.

Philpott, Stacy M., Brenda B. Lin, Shalene Jha, and Shannon J. Brines. 2008. “A Multi-Scale Assessment of Hurricane Impacts on Agricultural Landscapes Based on Land Use and Topographic Features.” *Agriculture, Ecosystems and Environment* 128 (1–2): 12–20. https://doi.org/10.1016/j.agee.2008.04.016.

Stewart, Ruth, Laurenz Langer, Natalie Rebelo Da Silva, Evans Muchiri, Hazel Zaranyika, Yvonne Erasmus, Nicola Randall, et al. 2015. “The Effects of Training, Innovation and New Technology on African Smallholder Farmers’ Economic Outcomes and Food Security: A Systematic Review.” *Campbell Systematic Reviews* 11 (1): 1–224. https://doi.org/10.4073/csr.2015.16.

Tazeze, Aemro, Jemma Haji, and Mengistu Ketema. 2012. “Climate Change Adaptation Strategies of Smallholder Farmers: The Case of Babilie District, East Harerghe Zone of Oromia Regional State of Ethiopia.” *Journal of Economics and Sustainable Development Www.Iiste.Org ISSN*. Vol. 3. Online. www.iiste.org.

Ville, Arlette S. Saint, Gordon M. Hickey, Uli Locher, and Leroy E. Phillip. 2016. “Exploring the Role of Social Capital in Influencing Knowledge Flows and Innovation in Smallholder Farming Communities in the Caribbean.” *Food Security* 8 (3): 535–49. https://doi.org/10.1007/s12571-016-0581-y.

**HH Questionnaire Overview**

| **Screening Questions** |
| --- |
| What is today's date? |
| Enumerators Name |
| Are the bananas on produced your farm certified as Organic? |
| Are the bananas produced on your farm certified as Fairtrade? |
| Are the bananas on produced your farm certified as Rainforest Alliance? |
| Are you the decision maker on the farm? |
|  |
| **Identification** |
| Region |
| District |
| Village |
| Name of respondent |
| Mobile phone number |
| Email |
| Gender |
| Age (in years) |
| What is your level of education? |
| How many people live in your household? |
|  |
| **Crop Plot Roster** |
| What is the total area of you farm including all plots? |
| How many plots do you have? |
| Number of plots |
| Which crops do you grow? |
| Total area? |
| Unit |
| How much did you produce last year? (Volume in Kgs) |
| What percentage of this did you sell of this? (%) |
| What was the average price you received for this crop? (RD$/kg) |
| Was this crop certified as organic? |
| Was your annual production of this crop reduced in 2017 because of flooding? If so, by how much? (%) |
|  |
|  |
| **Banana Plot Roster** |
| How many plots with bananas on do you have? |
| Do you have a packhouse? |
| Number of banana plots |
| Who owns the plot? |
| Who cultivates the plot? |
| How big is the banana plot? (Ha) |
| When did you plant the plants on this plot? |
| How many banana plants per terrea are planted in this plot? |
| Does this plot have access to irrigation? If so, what type? |
| Is this plot at risk of flooding from the river? |
| Which intercrops are present in this plot? |
| Do you have cableways installed on this plot? |
| What was your yield in 2018? |
| What was your yield in 2017? |
| What was your yield in 2016? |
| How many years ago was this plot certified as organic? |
| How far is the middle of this plot from the river? (m) |
|  |
|  |
| **Marketing and Sales** |
| What proportion of your sales of banana were through the co-operative? |
| How much did you make from banana sales in 2018? |
| What percentage of your annual production 2018 were you able to sell as export bananas? |
| What price did you get for export bananas 2018? |
| What price did you get for bananas sold on the local market in 2018? |
| How much did you make from banana sales in 2017? |
| What percentage of your annual production 2017 were you able to sell as export bananas? |
| What price did you get for export bananas 2017? |
| What price did you get for bananas sold on the local market in 2017? |
| How much did you make from banana sales in 2016? |
| What percentage of your annual production 2016 were you able to sell as export bananas? |
| What price did you get for export bananas 2016? |
| What price did you get for bananas sold on the local market in 2016? |
| Did you receive an extra financial premium per box of bananas for your certification? |
| If, so how much? (State certification and premium amount described) |
| NUMBER OF BUYERS? |
| Did you have a contract to sell bananas to a particular co-operative/buyer last year? If so, please summarise. |
|  |
| **Banana Farm Inputs** |
| Which inputs do you use on your farm? And how much do you use on your farm per year for bananas? |
| How much does this cost in total on your farm per year for bananas? |
|  |
| **Shock Experience** |
| have you ever experienced flooding? How many times did you experience flooding on your banana farm? (None) |
| Which was the worst flooding event in terms of impact to your farm and livelihood ? |
| What proportion of your total production was lost and how long did it take you to return to normal production? |
| Focusing now on flooding caused by Hurricane Maria in early September 2017: |
| How many terreas of your banana farm were flooded? |
| How many weeks did it take for the water to drain away completely? |
| What % of your banana production at that moment was lost? (Test ranges) |
| What % of your banana plants were destroyed? (Test ranges) |
| Was your packhouse damaged Maria? |
| What % of your cableways and drains were destroyed? (INFRASTRUCTURE GENERAL) |
| What percentage of your banana plants were damaged by the wind? (e.g. shredding of leaves) |
| Focusing now on flooding caused by Hurricane Irma in late September 2017: |
| How many terreas of your banana farm were flooded? |
| How many weeks did it take for the water to drain away completely? |
| What % of your banana production at that moment was (ACRES / TAREAS)? |
| What % of your banana plants were destroyed? |
| Was your packhouse damaged Irma? |
| What % of your cableways and drains were destroyed? |
| What percentage of your banana plants were damaged by the wind? (e.g. shredding of leaves) |
| What % of your annual banana production was lost because of the floods in 2017? |
| Did you lose any livestock as a result of the flooding in 2017 if so what was the value of this livestock? |
|  |
| **Pests and disease** |
| Do you experience any of these pests and diseases on your farm? |
| Did any of these pests and diseases increase after the flooding caused by Hurricane Maria? |
|  |
| **Shock Preparation** |
| Please list any measures you take to protect your banana production against flooding. (These can be physical, financial etc) |
| Do you have any form of insurance to protect your banana farm against flooding? |
|  |
| **Shock Response** |
| Please list any measures you take when you realise that flooding is about to occur or is occurring on your farm. |
|  |
| **Recovery** |
| What % of your banana farm did you have to replant after Hurricane Maria? |
| How much did it cost you per TAREA to replant? |
| What percentage of your plants did you have to cut for them to regrow after Maria? |
| How many weeks after the initial flooding did you judge your farm ready to be replanted? |
| How many weeks after the initial flooding did you start replanting? |
| Did you replant all the blocks at the same time? |
| If you did not replant all at the same time, why not? |
| How many plants per Ha did you replant your blocks at? |
| What machinery did you use? |
| How many weeks did it take to replant all of your farm that was damaged? |
| If anything, what restricted your pace of replanting? |
| What were the major costs in the replanting process? |
| Did you receive any support to replant your farm? |
| Did you add any additional inputs to the soil during land preparation? |
| Were you able to sell your new production after the hurricane flooding for export? |
| To reduce the effects of the flood shock and loss of your banana income on your livelihood what did you do? |
| What were the key constraints to you being able to return to your normal production and sales state? |
| When was your first sale of export bananas for the replanted blocks? |
|  |
| **Knowledge (can you make this knowledge more flooding relevant?)** |
| Do you know how to apply IPM on your farm? |
| Do you know how to design a drainage pattern to best serve your farm during a flood? |
| Do you know how to optimally space your banana plants for maximum yield? |
| Do you know how to produce biofermented compost for your farm? |
| Do you know how to use a computer? |
| Do you keep records of production and sales? |
| How many times have you received training on banana production in the last 2 years? |
| Have you had any training on how to protect your farm from flooding? |
| Have you had any training on how best to cultivate and replant your farm after a flood? |
| After the flooding caused by Hurricane Maria, did you receive any extra training to help you recover? |
|  |
| **Physical Capital** |
| Does your household own any of the following pieces of agricultural equipment? |
| Does your household own any livestock? Please list species and number. |
| Does your household own any of the following items? |
| As a result of the last flood did you have to sell any of the equipment, livestock, items listed above? Please list the item and how much you sold them for. |
|  |
| **Social Capital** |
| How much mobile phone credit do you use per month? |
| How many people do you discuss your banana production with in a normal month? |
| How many agricultural co-opertives (including banana) are you a member of ? |
| How many co-operative meetings do you attend in a normal year? |
| As a result of the last flood fid you reduce the number of agricultural co-operatives you are member of? OTHER |
|  |
| **Financial Capital** |
| What proportion of your household income does banana production make up? |
| Please list your other sources of agricultural income and the % they make up of your total HH income. |
| Does your household have a bank account? |
| Does your household have any savings? |
| Would your household be able to raise 30,000 Dominican pesos in the month after a flood to pay for the recovery of the banana farm? |
| How would you raise it? |
| After the flooding caused by Hurricane Maria, did you have to use more of your savings than usual? |
|  |
| **Natural Capital** |
| What proportion of your land owning is primary forest? |
| What proportion of your land owning is uncultivated secondary vegetation? |
| Is there any evidence of soil erosion on your farm? |
| What type of access do you have on your farm? |
| After the flooding caused by Hurricane Maria, did you notice any extra soil erosion on your farm? |
|  |
